# American Heart Association blood pressure classification and subsequent risk of preeclampsia

**DOI:** 10.1002/pmf2.70124

**Published:** 2025-10-22

**Authors:** Rebecca Horgan, Erkan Kalafat, Elena Sinkovskaya, Alfred Abuhamad, George Saade

**Affiliations:** ^1^ Macon & Joan Brock Virginia Health Sciences Old Dominion University Norfolk Virginia USA; ^2^ Deptartment of Obstetrics & Gynecology Koc University School of Medicine Istanbul Turkey; ^3^ Deptartment of Obstetrics & Gynecology University of Texas Medical Branch Galveston Texas USA

**Keywords:** American Heart Association, blood pressure, preeclampsia

## Abstract

**Background:**

In 2017, the American Heart Association (AHA) and American College of Cardiology updated their hypertension guidelines for the nonpregnant population, lowering the diagnostic thresholds to systolic blood pressure ≥130 mmHg or diastolic blood pressure ≥80 mmHg. This reclassification has raised important questions regarding whether blood pressure (BP) values previously considered within the normal range in pregnancy may, in fact, confer an increased risk for hypertensive disorders of pregnancy (HDPs).

**Objective:**

To evaluate the association between first‐trimester BP classified by AHA thresholds and the subsequent development of HDPs.

**Study design:**

This was a secondary analysis of a prospective, multicenter cohort study enrolling singleton pregnancies at ≤13 6/7 weeks’ gestation. Participants with preexisting hypertension were excluded. First‐trimester BP was categorized using AHA guidelines: normal (<120/80 mmHg), elevated (120–129/<80 mmHg), Stage 1 hypertension (130–139/80–89 mmHg), and Stage 2 hypertension (≥140/90 mmHg). The primary outcome was preeclampsia; secondary outcomes included gestational hypertension and HDPs. Associations were evaluated using multivariable Cox regression adjusted for maternal age, body mass index, parity, race, smoking status, and preeclampsia risk factors. Predictive performance was assessed using concordance indices.

**Results:**

Among 576 participants, 13.0% developed HDP, including 7.6% with preeclampsia. Incidence of preeclampsia increased stepwise with AHA BP category: 4.8% (normal), 11.2% (elevated), 14.6% (Stage 1), and 16.7% (Stage 2) (*p* < 0.001). Compared to normal BP, adjusted hazard ratios for preeclampsia were 2.24 (95% confidence interval [CI], 1.03–4.86) for elevated BP, 2.80 (95% CI, 1.38–5.67) for Stage 1, and 2.48 (95% CI, 0.32–19.01) for Stage 2. AHA‐based models outperformed American College of Obstetricians and Gynecologists (ACOG) thresholds (≥140/90 mmHg) in predicting HDP (*C*‐index, 0.71 vs. 0.65; *p* < 0.05).

**Conclusion:**

First‐trimester BP classification using AHA guidelines offers a more nuanced approach to stratifying HDP risk in pregnancy, enabling earlier identification of at‐risk individuals beyond the current ACOG definition of chronic hypertension in pregnancy, and supporting more personalized counseling and preventive strategies.

## 1 INTRODUCTION

Preeclampsia remains one of the leading causes of maternal and perinatal morbidity and mortality worldwide, contributing to adverse pregnancy outcomes such as fetal growth restriction, preterm birth, maternal seizure, stroke, and death [1, 2, 3, 4, 5, 6]. Early identification of individuals at increased risk is critical, as preventative strategies, including low‐dose aspirin, have been shown to reduce the incidence and severity of disease when initiated before 16 weeks’ gestation [7]. Early identification of individuals at increased risk for preeclampsia remains a challenge in obstetric care.

Traditionally, chronic hypertension in pregnancy has been defined as systolic blood pressure (SBP) ≥140 mmHg or diastolic blood pressure (DBP) ≥90 mmHg diagnosed before pregnancy or prior to 20 weeks' gestation. However, in 2017, the American Heart Association (AHA) and American College of Cardiology updated their hypertension guidelines for the nonpregnant population, lowering the diagnostic thresholds to SBP ≥ 130 mmHg or DBP ≥ 80 mmHg, and introducing new categories: elevated BP (120–129/<80 mmHg), Stage 1 hypertension (130–139/80–89 mmHg), and Stage 2 hypertension (≥140/90 mmHg) [8]. This reclassification has raised important questions regarding whether blood pressure (BP) values previously considered within the normal range in pregnancy may, in fact, confer an increased risk for hypertensive disorders of pregnancy (HDPs), particularly preeclampsia [9, 10, 11].

While the American College of Obstetricians and Gynecologists (ACOG) has not adopted these thresholds in obstetric care, emerging evidence suggests that elevated BP or Stage 1 hypertension, as per AHA guidelines, may increase the risk of HDPs and preeclampsia [12, 13, 14, 15, 16]. However, prior studies have reported conflicting findings, and the available data are predominantly retrospective in nature [12, 13, 14, 17, 18]. Therefore, whether AHA‐classified BP in early pregnancy provides clinically useful risk stratification for preeclampsia, beyond traditional definitions, remains uncertain.

To address this gap, we conducted a secondary analysis of a prospective, multicenter cohort study to evaluate the association between first‐trimester BP classified by AHA criteria and the subsequent risk of preeclampsia and other HDPs. We hypothesized that the AHA classification would more accurately identify individuals at increased risk than the current ACOG cutoff of 140/90 mmHg.

### 1.1 Study design

This was a secondary analysis of a prospective, longitudinal cohort study funded by the National Institutes of Health on the human placenta. The aim of the parent study was to apply novel ultrasound tools, to study placental development in pregnancy, and to create a multivariate model to predict preterm birth. Pregnant individuals aged 18–50 years with singleton gestations were recruited at ≤13 6/7 weeks’ gestation from two academic centers. Exclusion criteria included any fetal or placental abnormality at the time of recruitment in the first trimester. Abnormal placental implantation and umbilical cord abnormalities were defined as placenta previa (covering the internal cervical os), bilobed placenta, and marginal or velamentous cord insertion. For this secondary analysis, patients with a prepregnancy diagnosis of chronic hypertension were also excluded. Chronic hypertension was defined as SBP > 140 mmHg or DBP > 90 mmHg. Each participant in the cohort underwent eight longitudinal ultrasounds throughout pregnancy with delivery and outcome data obtained by trained research coordinators. Written informed consent was obtained from all participants, and the study was approved by the institutional review boards of both participating institutions.

At the first‐trimester study visit, BP was measured using an automated machine with appropriate sized cuff and categorized according to the 2017 AHA guidelines: normal (<120/80 mmHg), elevated (120–129/<80 mmHg), Stage 1 hypertension (130–139/80–89 mmHg), and Stage 2 hypertension (140–180/90–120 mmHg) for this analysis [8]. BP measurements were obtained by trained research staff blinded to clinical outcomes, and all pregnancy management decisions were made independently by the clinical care team. The primary outcome was preeclampsia, defined per ACOG criteria [19]. Secondary outcomes included gestational hypertension and the broader category of HDPs [19].

Associations between AHA BP categories and risk of preeclampsia and HDP were assessed using Cox proportional hazards models, adjusted for maternal age, body mass index (BMI), parity, race, smoking status, and the presence of preeclampsia risk factors. Cumulative incidence functions were generated to assess time‐to‐onset by gestational age. Mean arterial pressure (MAP) was analyzed as a continuous predictor using restricted cubic spline modeling. Model discrimination was evaluated using concordance indices to compare the predictive performance of AHA versus traditional ACOG BP thresholds (≥140/90 mmHg). A *p*‐value <0.05 was considered statistically significant.

## 2 RESULTS

**FIGURE 1:**
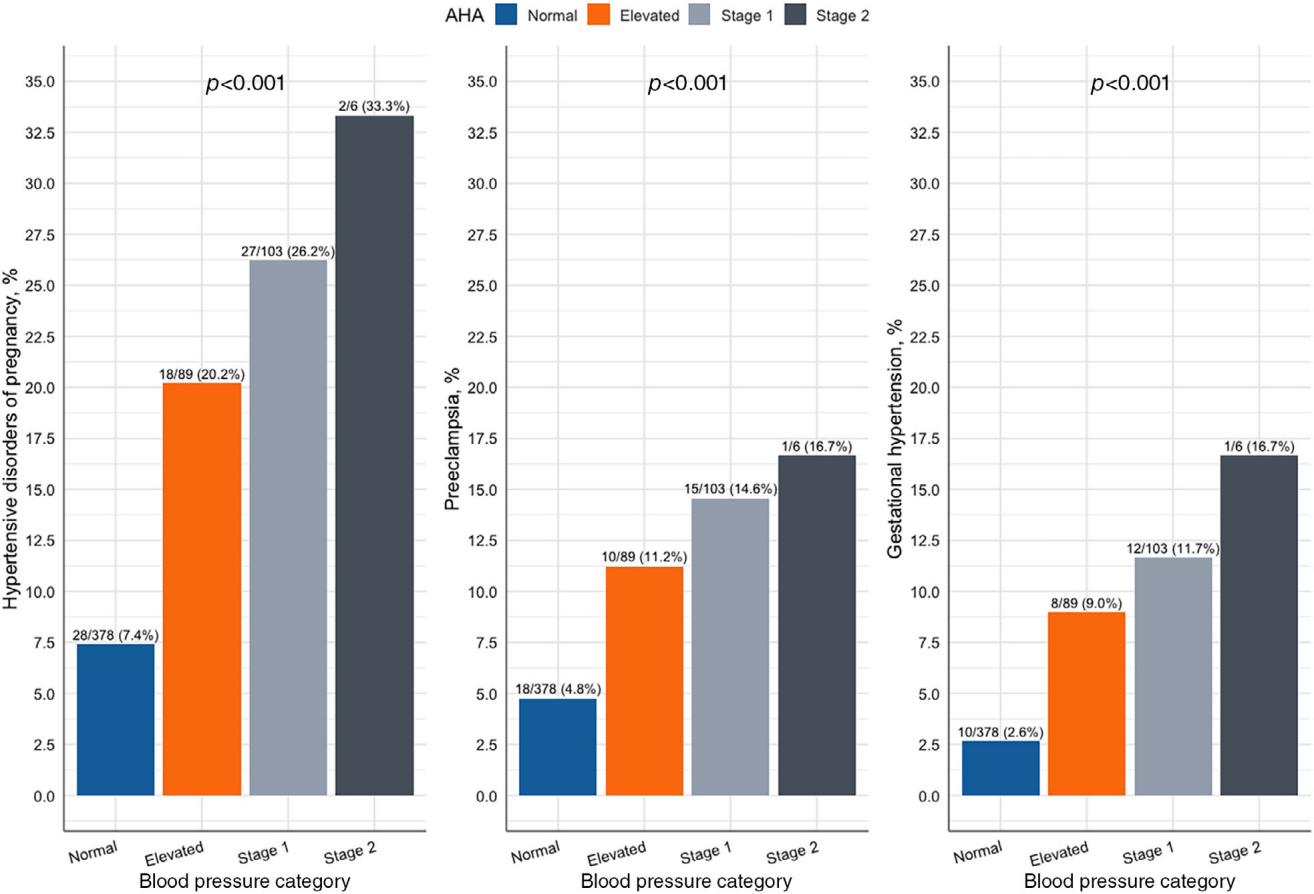
Association between blood pressure categories based on American Heart Association (AHA) classification and risk of hypertensive disorders of pregnancy, preeclampsia, and gestational hypertension.

There were 610 patients in the original cohort. A total of 576 (94%) patients met eligibility criteria and were included in this analysis. Baseline characteristics and outcomes based on BP categorization in the first trimester are presented in Table 1. In the first trimester, 65.6% of patients had normal BP, 15.5% had elevated BP, 17.5% had Stage 1 hypertension, and 1.0% had Stage 2 hypertension, based on AHA classification. Among the 576 patients, 75 (13.0%) developed an HDP, including 44 (7.6%) with preeclampsia and 31 (5.4%) with gestational hypertension. Compared to those with normal first‐trimester BP, individuals with elevated BP, Stage 1 hypertension, and Stage 2 hypertension experienced progressively higher risks of HDPs (Figure 1). The incidence of preeclampsia increased stepwise across AHA BP categories: 4.8% for normal BP, 11.2% for elevated BP, 14.6% for Stage 1 hypertension, and 16.7% for Stage 2 hypertension (*p* < 0.001) (Figure 1). A similar pattern was observed for gestational hypertension, occurring in 2.6%, 9%, 11.7%, and 16.7% of patients, respectively (*p* < 0.001) (Figure 1). Compared to individuals with normal first‐trimester BP, those with elevated BP, Stage 1 hypertension, and Stage 2 hypertension had a significantly increased risk of both preeclampsia and HDPs, even after adjustment for maternal age, BMI, race, parity, and prenatal risk factors (Tables 2 and 3).

**TABLE 1:** Baseline characteristics and outcomes according to American Heart Association (AHA) hypertension categories.

	Normal (*N* = 378)	Elevated (*N* = 89)	Hypertension Stage 1 (*N* = 103)	Hypertension Stage 2 (*N* = 6)	*p* ^a^
Age					
Median (IQR)	27.0 (23.0–31.0)	26.0 (22.0–31.0)	27.0 (22.5–30.5)	31.5 (26.5–34.2)	0.492
Race					
White	190 (50.3)	48 (53.9)	54 (52.4)	3 (50.0)	0.917
Black	176 (46.6)	38 (42.7)	46 (44.7)	3 (50.0)	
Asian	5 (1.3)	2 (2.2)	2 (1.9)	0 (0.0)	
Native American	2 (0.5)	0 (0.0)	0 (0.0)	0 (0.0)	
Other	5 (1.3)	1 (1.1)	1 (1.0)	0 (0.0)	
Employment					
None	171 (45.2)	38 (42.7)	44 (42.7)	1 (16.7)	0.529
Part‐time	42 (11.1)	7 (7.9)	10 (9.7)	0 (0.0)	
Full‐time	139 (36.8)	35 (39.3)	39 (37.9)	5 (83.3)	
Unknown	26 (6.9)	9 (10.1)	10 (9.7)	0 (0.0)	
Insurance					
Self‐pay	3 (0.8)	3 (3.4)	1 (1.0)	0 (0.0)	0.479
Private insurance	106 (28.0)	23 (25.8)	30 (29.1)	3 (50.0)	
Government insurance	260 (68.8)	63 (70.8)	71 (68.9)	3 (50.0)	
Unknown	9 (2.4)	0 (0.0)	1 (1.0)	0 (0.0)	
Parity					
Multiparous	259 (68.5)	53 (59.6)	62 (60.2)	3 (50.0)	0.184
Primiparous	119 (31.5)	36 (40.4)	41 (39.8)	3 (50.0)	
History of preeclampsia					
No	367 (97.1)	84 (94.4)	93 (90.3)	5 (83.3)	0.014
Yes	11 (2.9)	5 (5.6)	10 (9.7)	1 (16.7)	
BMI					
Median (IQR)	24.1 (22.0–27.8)	26.1 (22.5–29.0)	26.9 (23.4–30.0)	26.8 (25.1–32.5)	<0.001
First‐trimester systolic BP					
Median (IQR)	108.5 (103.0–113.0)	123.0 (121.0–126.0)	127.0 (121.0–132.0)	137.0 (133.0–141.8)	<0.001
First‐trimester diastolic BP					
Median (IQR)	68.0 (63.0–72.0)	74.0 (68.0–77.0)	81.0 (80.0–84.0)	92.0 (86.0–92.8)	<0.001
Hypertensive disorder of pregnancy	28 (7.4)	18 (20.2)	27 (26.2)	2 (33.3)	<0.001
Preeclampsia	18 (4.8)	10 (11.2)	15 (14.6)	1 (16.7)	0.003
Gestational hypertension	10 (2.6)	8 (9.0)	12 (11.7)	1 (16.7)	0.001

Abbreviations: BMI, body mass index; BP, blood pressure; IQR, interquartile range.

^a^ Kruskal–Wallis or chi‐squared test.

**TABLE 2:** Multivariable Cox regression analysis for factors associated with preeclampsia.

Predictors	Hazard ratio	95% CI	*p* ^a^
AHA blood pressure category			
Normal	Reference		
Elevated	2.24	1.03–4.86	**0.042**
Stage 1	2.80	1.38–5.67	**0.004**
Stage 2	2.48	0.32–19.01	0.381
Race			
White	Reference		
Black	2.33	1.25–4.34	**0.008**
Other	NE	–	–
Age	1.02	0.96–1.08	0.491
Parity			
Nulliparous	Reference		
Multiparous	0.50	0.25–0.99	**0.047**
BMI	1.02	0.96–1.08	0.536
Risk factors for preeclampsia			
Absent	Reference		
Present	2.81	1.00–7.91	0.050

Abbreviations: AHA, American Heart Association; BMI, body mass index; CI, confidence interval; NE, not estimable.

Bold values denote the statistical significance.

^a^ Multivariable Cox regression.

**TABLE 3:** Multivariable Cox regression analysis for factors associated with hypertensive disorder of pregnancy (HDP).

Predictors	Hazard ratio	CI	*p* ^a^
AHA blood pressure category			
Normal	Reference		
Elevated	2.61	1.44–4.72	**0.002**
Stage 1	3.28	1.91–5.63	**<0.001**
Stage 2	3.29	0.77–14.07	0.109
Race			
White	Reference		
Black	1.35	0.85–2.15	0.207
Other	NE	–	–
Age	1.01	0.97–1.06	0.531
Parity			
Nulliparous	Reference		
Multiparous	0.62	0.37–1.04	0.069
BMI	1.04	0.99–1.09	0.082
Risk factor for preeclampsia			
Absent	Reference		
Present	3.61	1.70–7.66	**0.001**

Abbreviations: AHA, American Heart Association; BMI, body mass index; CI, confidence interval; NE, not estimable.

Bold values denote the statistical significance.

^a^ Multivariable Cox regression.

**FIGURE 2:**
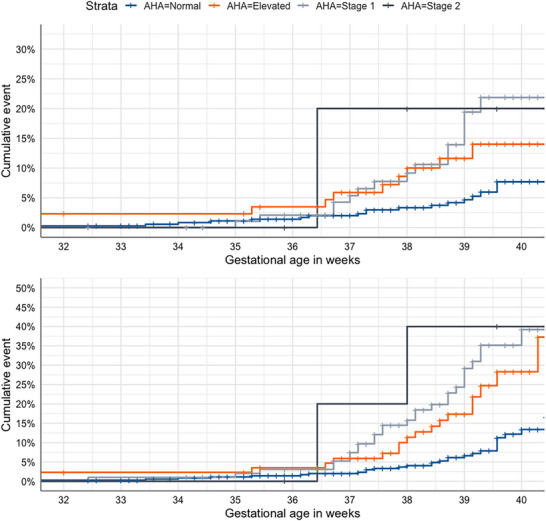
Cumulative incidence of preeclampsia (top) and hypertensive disorder of pregnancy (HDP) (bottom) by gestational age and blood pressure categories. AHA, American Heart Association.

**FIGURE 3:**
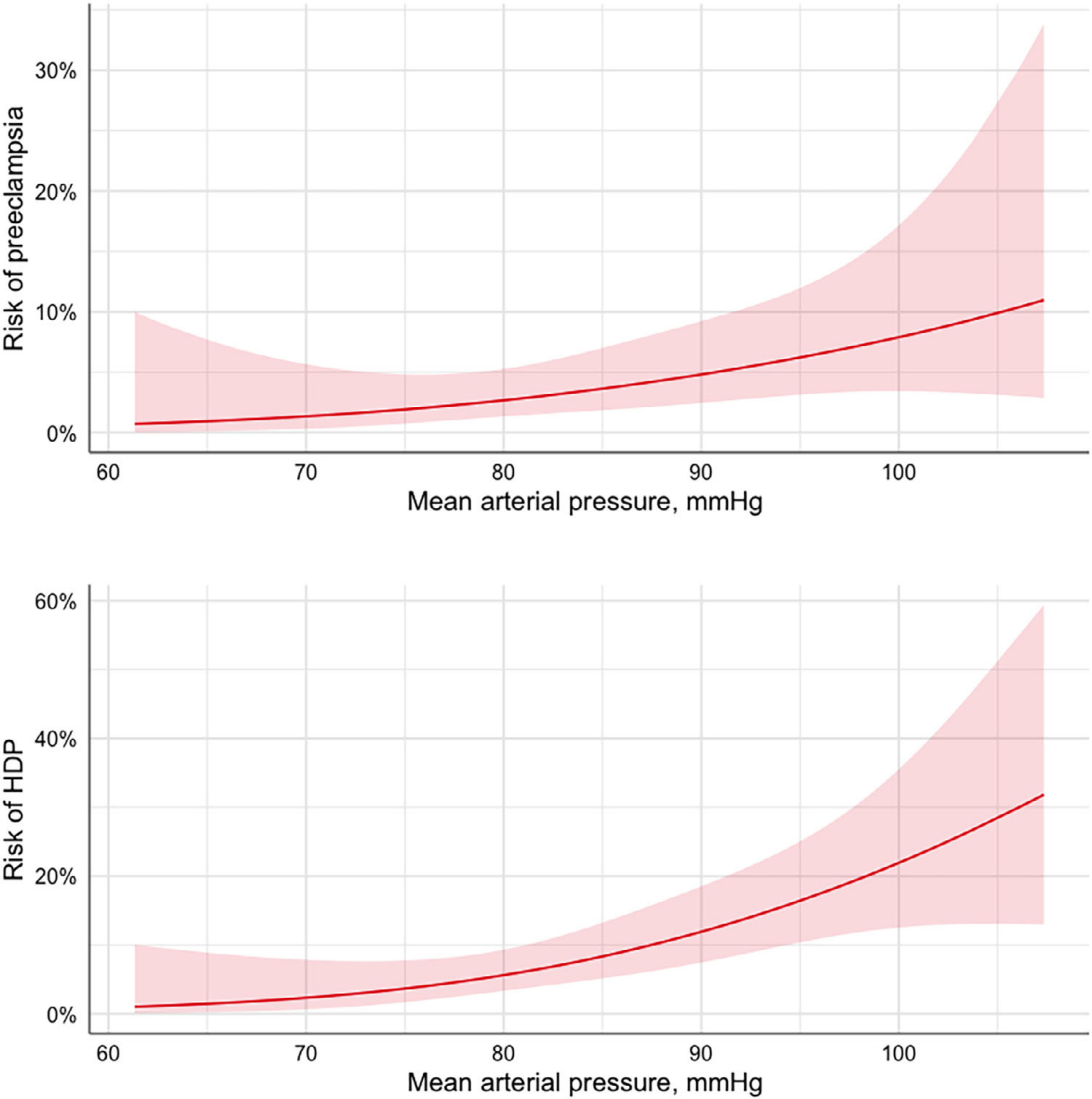
Risk of preeclampsia and hypertensive disorder of pregnancy (HDP) according to mean arterial pressure in the first trimester.

Individuals with elevated BP, Stage 1 hypertension, and Stage 2 hypertension developed preeclampsia and HDPs both earlier in gestation and at higher cumulative rates compared to those with normal BP (Figure 2). Consistent with these findings, adjusted risk curves revealed a continuous, dose‐dependent relationship between first‐trimester MAP and the risk of preeclampsia and HDPs, with risk rising progressively across the MAP spectrum (Figure 3). The Cox regression model using AHA classification had a significantly higher concordance index compared to the model using the current ACOG classification of prepregnancy hypertension using a singular 140 mmHg systolic or 90 mmHg diastolic cutoff for defining chronic hypertension before 20 weeks.

## 3 DISCUSSION

Our study demonstrates that first‐trimester BP classified according to AHA guidelines is independently associated with later development of preeclampsia. We found that pregnant individuals with elevated BP, Stage 1 hypertension, and Stage 2 hypertension in early pregnancy exhibited an increasing risk of developing preeclampsia compared to those with normal BP. Importantly, this association persisted even after adjusting for maternal age, BMI, race, parity, and smoking status. These findings support the hypothesis that even mildly elevated BP in early pregnancy reflects underlying vascular dysfunction that predisposes individuals to preeclampsia. Notably, we observed that the AHA classifications provided superior risk‐assessment capability compared to the ACOG hypertension diagnosis based on a 140/90 mmHg cutoff. These findings highlight the potential utility of the AHA guidelines in stratifying preeclampsia risk earlier in pregnancy.

Our findings align with emerging evidence suggesting that first‐trimester BP below the traditional 140/90 mmHg threshold may still be clinically significant. Several retrospective studies have observed an increased risk of preeclampsia among pregnant individuals with BP in early pregnancy of 120–139 mmHg systolic or 80–89 mmHg diastolic [12, 13, 20]. However, prior investigations were limited by retrospective study designs and inconsistent BP measurement protocols, which may have contributed to the variability in reported associations. Our findings are in contrast to a previous secondary analysis of a prospective study in the United Kingdom, which did not find lower BP cutoffs helpful for stratifying risk of preeclampsia [14]. This is possibly due to the differing patient populations as the UK study population was 75% Caucasian, whereas our study population was racially diverse and included a higher proportion of individuals with preexisting cardiovascular risk factors such as obesity [14]. Therefore, our cohort may have been more sensitive to lower BP cutoffs as a predictor of preeclampsia.

Our findings are consistent with and build upon growing evidence that early pregnancy BP, even within ranges previously considered normal in pregnancy, is a significant predictor of adverse outcomes. A recent meta‐analysis by Slade et al., incorporating over 700,000 pregnancies, concluded that both elevated BP and Stage 1 hypertension in early pregnancy are associated with a significantly increased risk of preeclampsia [10]. Similarly, Hauspurg et al. used data from the prospective nuMoM2b cohort of 8899 nulliparous individuals recruited between 2010 and 2014 to evaluate the relationship between AHA BP categories and HDPs [20]. They found that Stage 1 hypertension in early pregnancy was associated with a nearly threefold increased risk of preeclampsia (adjusted Relative Risk [aRR] 2.92, 95% Confidence Interval [CI] 1.44–5.95) and a more than twofold increased risk of gestational hypertension (aRR 2.36, 95% CI 1.37–4.06) [20]. While robust, their analysis was limited to nulliparous women and had lower racial diversity. In contrast, our prospective, racially diverse, multicenter cohort included both nulliparous and multiparous individuals, increasing generalizability. We similarly found that Stage 1 hypertension in the first trimester was associated with a significantly increased risk of preeclampsia (adjusted hazard ratio [aHR] 3.02, 95% CI 1.49–6.12) and HDPs (aHR 2.63, 95% CI 1.40–4.94). Moreover, we extended the evidence base by incorporating time‐to‐event methods and cumulative incidence analyses by gestational age, demonstrating earlier and more frequent onset of disease in individuals with elevated BP or Stage 1 hypertension. Finally, we used concordance indices to show that the AHA classification provided superior risk prediction compared to the traditional ACOG threshold of 140/90 mmHg. Collectively, our results reinforce and expand upon prior findings, supporting the clinical utility of AHA‐defined BP thresholds for early pregnancy risk stratification in a broader obstetric population.

Our findings suggest that first‐trimester BP should be more carefully considered in risk assessment for HDPs. Under current ACOG guidelines, only individuals with BP ≥140/90 mmHg before 20 weeks' gestation are classified as having chronic hypertension [21]. However, our findings indicate that lower thresholds, such as those defined by the AHA, offer clinically meaningful risk discrimination. Notably, all AHA categories outside of the normal range were associated with a >10% risk of preeclampsia, meeting the U.S. Preventive Services Task Force (USPSTF) threshold for recommending low‐dose aspirin prophylaxis [22]. These findings have important implications for early pregnancy counseling, risk stratification, and clinical management. However, before adopting changes in clinical practice, such as routine aspirin initiation for patients with elevated or Stage 1 BP in early pregnancy, prospective interventional studies are needed to determine whether such strategies reduce the incidence of preeclampsia in this population. Additionally, integrating biomarkers of endothelial dysfunction may help elucidate the mechanistic links between early gestation BP and preeclampsia pathogenesis. Another important nuance in our findings is the timing of preeclampsia onset by first‐trimester BP category. Our cumulative incidence analysis (Figure 2) demonstrates that much of the excess risk in individuals with elevated BP or Stage 1 hypertension reflects the development of term preeclampsia. This distinction has direct implications for preventive strategies. Current evidence supports low‐dose aspirin in reducing the incidence of preterm preeclampsia when initiated before 16 weeks’ gestation, but randomized trials have not shown a clear benefit for prevention of term disease [7, 22]. Thus, while our results indicate that patients with AHA‐defined BP elevations meet the USPSTF risk threshold for aspirin prophylaxis, it is possible that aspirin may not substantially reduce the burden of term‐onset disease in this group.

A major strength of this study is its prospective design, which allowed for systematic BP classification and longitudinal follow‐up with robust collection of data by trained research coordinators and ascertainment of outcomes. In addition, our study population represented a diverse demographic profile. However, some limitations should be acknowledged. BP measurements were taken at a single time point in the first trimester, which may not fully capture longitudinal BP trends throughout pregnancy. In addition, patients with a prior diagnosis of chronic hypertension were excluded because the parent cohort did not capture first‐trimester antihypertensive use, which could have artificially lowered measured BPs. Consequently, only a small proportion of participants in our study met criteria for Stage 2 hypertension. The lack of prepregnancy BP measurements also raises the possibility that individuals classified as normotensive or elevated based on first‐trimester values may have had higher BPs prior to conception, particularly given that early pregnancy is associated with a physiological decline in BP of approximately 5–10 mmHg [23].

First‐trimester BP classification using AHA guidelines provides a valuable tool for stratifying preeclampsia risk, beyond the current ACOG definition of chronic hypertension in pregnancy. These findings support the consideration of AHA guidelines in maternal care and could be useful in developing preventative strategies for high‐risk pregnancies. Further research is needed to explore the integration of this approach with other predictive markers.

## CONFLICT OF INTEREST STATEMENT

The authors declare no conflicts of interest.

## Data Availability

The data that support the findings of this study are available from the corresponding author upon reasonable request.
